# Inhibition of nitric oxide in LPS-stimulated macrophages of young and senescent mice by δ-tocotrienol and quercetin

**DOI:** 10.1186/1476-511X-10-239

**Published:** 2011-12-20

**Authors:** Asaf A Qureshi, Xiaoyu Tan, Julia C Reis, Mostafa Z Badr, Christopher J Papasian, David C Morrison, Nilofer Qureshi

**Affiliations:** 1 Department of Basic Medical Sciences, University of Missouri-Kansas City, 2411 Holmes Street, Kansas City, MO 64108, USA; 2 Department of Medicine, University of Kansas Medical Center, 3901 Rainbow Boulevard, Kansas City, KS 66160, USA; 3 Division of Pharmacology and Toxicology, School of Pharmacy, University of Missouri-Kansas City, 2464 Charlotte Street, Kansas City, MO 64108, USA

## Abstract

**Background:**

Changes in immune function believed to contribute to a variety of age-related diseases have been associated with increased production of nitric oxide (NO). We have recently reported that proteasome inhibitors (dexamethasone, mevinolin, quercetin, δ-tocotrienol, and riboflavin) can inhibit lipopolysaccharide (LPS)-induced NO production *in vitro *by RAW 264.7 cells and by thioglycolate-elicited peritoneal macrophages derived from four strains of mice (C57BL/6, BALB/c, LMP7/MECL-1^-/- ^and PPAR-α^-/- ^knockout mice). The present study was carried out in order to further explore the potential effects of diet supplementation with naturally-occurring inhibitors (δ-tocotrienol and quercetin) on LPS-stimulated production of NO, TNF-α, and other pro-inflammatory cytokines involved in the ageing process. Young (4-week-old) and senescent mice (42-week old) were fed control diet with or without quercetin (100 ppm), δ-tocotrienol (100 ppm), or dexamethasone (10 ppm; included as positive control for suppression of inflammation) for 4 weeks. At the end of feeding period, thioglycolate-elicited peritoneal macrophages were collected, stimulated with LPS, LPS plus interferon-β (IFN-β), or LPS plus interferon-γ (IFN-γ), and inflammatory responses assessed as measured by production of NO and TNF-α, mRNA reduction for TNF-α, and iNOS genes, and microarray analysis.

**Results:**

Thioglycolate-elicited peritoneal macrophages prepared after four weeks of feeding, and then challenged with LPS (10 *ng *or 100 *ng*) resulted in increases of 55% and 73%, respectively in the production of NO of 46-week-old compared to 8-week-old mice fed control diet alone (respective control groups), without affecting the secretion of TNF-α among these two groups. However, macrophages obtained after feeding with quercetin, δ-tocotrienol, and dexamethasone significantly inhibited (30% to 60%; ***P ***< 0.02) the LPS-stimulated NO production, compared to respective control groups. There was a 2-fold increase in the production of NO, when LPS-stimulated macrophages of quercetin, δ-tocotrienol, or dexamethasone were also treated with IFN-β or IFN-γ compared to respective control groups. We also demonstrated that NO levels and iNOS mRNA expression levels were significantly higher in LPS-stimulated macrophages from senescent (0.69 vs 0.41; ***P ***< 0.05), compared to young mice. In contrast, age did not appear to impact levels of TNF-α protein or mRNA expression levels (0.38 vs 0.35) in LPS-stimulated macrophages. The histological analyses of livers of control groups showed lesions of peliosis and microvesicular steatosis, and treated groups showed Councilman body, and small or large lymphoplasmacytic clusters.

**Conclusions:**

The present results demonstrated that quercetin and δ-tocotrienols inhibit the LPS-induced NO production *in vivo*. The microarray DNA analyses, followed by pathway analyses indicated that quercetin or δ-tocotrienol inhibit several LPS-induced expression of several ageing and pro-inflammatory genes (IL-1β, IL-1α, IL-6, TNF-α, IL-12, iNOS, VCAM1, ICAM1, COX2, IL-1RA, TRAF1 and CD40). The NF-κB pathway regulates the production of NO and inhibits the pro-inflammatory cytokines involved in normal and ageing process. These *ex vivo *results confirmed the earlier *in vitro *findings. The present findings of inhibition of NO production by quercetin and δ-tocotrienol may be of clinical significance treating several inflammatory diseases, including ageing process.

## Background

In recent years, the concept that age-associated diseases (e.g. cancer, cardiovascular disease, dementia) might be attributable, in part, to dysregulated inflammatory responses has been the subject of extensive discussion [1]. We have studied the host inflammatory response to endotoxin (lipopolysaccharide = LPS) for many years, and recently have focused on the role of the proteasomes in regulating LPS-induced inflammatory responses in various systems [2,3]. Consequently, we were intrigued by a report that LPS-stimulated macrophages from senescent (22-month-old) mice produce approximately 10 times more nitric oxide (NO) than similarly stimulated macrophages from 2-month-old mice [4], as we have demonstrated that proteasome inhibitors can suppress NO production by murine macrophages [3]. Specifically, we have identified a variety of naturally-occurring proteasome inhibitors that have the capacity to suppress LPS-induced production of NO and secretion of TNF-α, as well as signaling pathways leading to production of TNF-α and other pro-inflammatory cytokines in RAW 264.7 cells, and thioglycolate-elicited peritoneal macrophages derived from four strains of mice [C57BL/6, BALB/c, double knockout LMP7/MECL-1^-/-^, and peroxisome proliferator-activated receptor-α^-/- ^(PPAR-α^-/-^) knockout mice] [3]. As several of these naturally-occurring compounds appear to be non-toxic, we have become intrigued with the concept of diet supplementation with these agents, with the ultimate goal of preventing some of the damage attributable to dysregulated inflammatory responses associated with ageing. We have been greatly encouraged by our recent results demonstrating that serum TNF-α and NO levels were significantly reduced in chickens fed diets supplemented with either quercetin or δ-tocotrienol, two naturally-occurring proteasome inhibitors [5].

**Figure 1:**
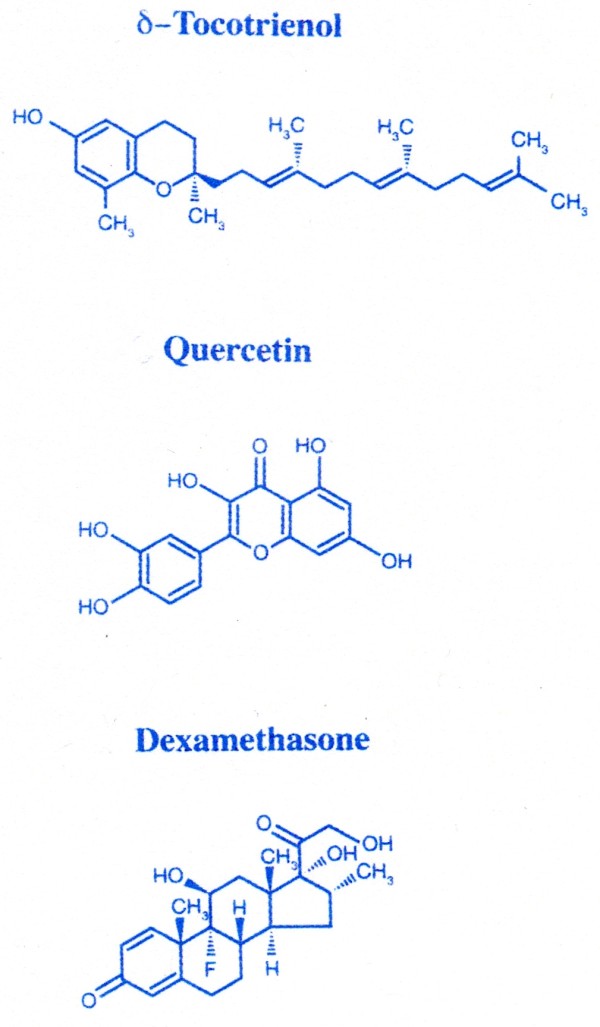
**Chemical structures of various compounds used in this study**.

The main objective of the present study was to expand upon our previous *in vitro *studies with mice and *in vivo *studies with chickens [3,5]. Specifically, we were interested in evaluating the anti-inflammatory properties of dietary supplementation with quercetin and δ-tocotrienol *in vivo *in mice; dexamethasone, a well known anti-inflammatory agent was used as a positive control. Control diet or diets supplemented with quercetin, δ-tocotrienol, or dexamethasone were fed to young (4-week-old) and senescent (42-week-old) male C57BL/6 mice for 4 weeks (Figure 1). Thioglycolate-elicited peritoneal macrophages were then collected from mice, stimulated with LPS, and the capacity of these macrophages to generate inflammatory responses (e.g. TNF-α secretion and NO production, TNF-α and iNOS gene expression, microarray analysis) was assessed under a variety of conditions. The results of the current study suggest that these compounds could be used to prevent or slow down the symptoms (fatigue, loss of memory and weakness of body muscles) associated with the ageing process in humans.

## Materials and methods

### Materials

Highly purified, deep rough chemotype LPS (Re LPS) from *E. coli *D31m4 was prepared as described by Qureshi et al. [6]. Thioglycolate and dexamethasone were purchased from Sigma-Aldrich (St. Louis, MO) and RNeasy mini kit from QIAGEN Sciences (Germantown, MD, USA). Quercetin was purchased from Alfa Aesar (Johnson Matthey Co. Lancaster, UK) and 50% δ-tocotrienol was received as a gift from American River, MA, USA.

### Animals

All mice used in this study received humane care in compliance with principles of laboratory animals care formulated by the National Society of Health Guide for the Care and Use of Laboratory Animals by the US National Institute of Health (NIH publication No 85-23, revised 1996). The experimental procedures involving animals were carefully reviewed and approved by the Institutional Animal Care and Use Committee of UMKC, MO, USA.

The 3-week-old and 41-week-old C57BL/6 male mice were obtained from "The Jackson Laboratory" (Bar Harbor, ME, USA), and acclimatized to the new environment for seven days before the start of experimentation. Mice were fed regular commercial mice diet with or without supplements (see below) ad libitum and had free access to water throughout the experiment. A 12 h light (08 am) and 12 h dark (08 pm) cycle was maintained during the feeding period. The study was carried out under a FDA IND number 36906 (USA).

### Purification of δ-tocotrienol from purified 50% fraction of annatto seeds

A commercial 50% purified fraction of δ-tocotrienol was obtained from American River (Boston, MA), and subjected to further purification as previously described [7]. The purity of δ-tocotrienol was established by high pressure liquid chromatography (HPLC) against its standards as reported earlier [8].

### Diet preparation and supplementation

Four groups of 3-week-old C57BL/6 male (3 mice/group; *n *= 12) and 41-week-old C57BL/6 (*n *= 12) were acclimatized for seven days to their new environment. Mice (4-week-old and 42-week-old) were fed mice commercial diet (CD; control group), or CD supplemented with quercetin (100 ppm), δ-tocotrienol (100 ppm) or dexamethasone (10 ppm) for 4 weeks. For diet supplementation quercetin (200 mg), δ-tocotrienol (200 mg) or dexamethasone (20 mg) were dissolved in 100 mL of ethanol and added to 2 kg diet. The diet of the control group was also mixed with 100 mL of ethanol. All mice were weighed individually at the start and at the end of the experiment.

Commercial diet (2 kg) pellets were crushed to a course powder and mixed with 100 mL ethanol alone, or 100 mL ethanol containing quercetin (100 ppm), δ-tocotrienol (100 ppm) or dexamethasone (10 ppm); ethanol was evaporated during mixing. Food and water was provided to mice ad libitum.

### Collection and LPS-stimulation of peritoneal macrophages

After being fed control diet or supplemented diets for 4 weeks, thioglycolate-elicited peritoneal macrophages were prepared from each mouse as described previously [9,10]. Thioglycolate-elicited peritoneal macrophages (1 × 10^7^/well) prepared from each group were challenged with LPS alone (10 or 100 *ng*/well), LPS (10 *ng*/well) plus IFN-β (50 U/well), LPS (10 *ng*/well) plus IFN-γ (50 U/well), or medium alone (control group), and incubated at either room temperature (NO), or 37°C (TNF-α). After 4 h (LPS alone for the estimation of TNF-α), or 18 hours (for NO assay, LPS alone or LPS plus IFN-β or LPS plus IFN-γ ) stimulated cells were centrifuged at 2,000 rpm for 20 min. The cells were then harvested for RNA extraction, and the supernatants were stored in glass vials at -70°C for subsequent measures of nitric oxide (NO) and TNF-α levels.

### Histological studies of liver samples after feeding various compounds to 4-week-old or 42-week-old C57BL/6 male mice for 4 weeks

After removing the thiogltcolate-elicited peritoneal macrophages, mice were sacrificed and liver samples were removed, stored in 10% formalin at -70°C until histological analyses were carried out. After the tissues were fixed in formalin, they were embedded with paraffin and cut in the sagital plane. The sections were stained by hematoxylin and eosin and examined by light microscopy. A semi-quantitative evaluation of histological analyses of these liver samples were carried out as described previously [11]. Evaluation of samples were carried out by two pathologists, unaware of the treatments. Mean scores were assigned to each sample, scored ranged from 0 (normal appearance) to 40 (very severe impact). The means of the assigned values for each group were reported.

### Measurement of nitric oxide (NO) and TNF-α levels

NO levels were determined in thawed supernatants by measuring the amount of nitrite, a stable metabolic product of nitric oxide according to the reported procedure [12]. The assay mixture contained medium (100 μL) plus Griess reagent (100 μL) in round-bottom 96-well tissue culture plates, and absorption was measured at 570 nm using a "Microplate Reader" (MR 5000; Dynatech Labs, Inc. USA). The amount of nitrite was determined by comparison of unknowns using a NaNO2 standard curve. The NO detection limit is 0.20 nM [12].

TNF-a levels in thawed supernatants were determined using the Quantikine M ELISA kit (R&D System, Minneapolis, MN, USA) according to manufacturer's instructions. The lower limit of detection for TNF-a in this method is approximately, 5.0 *pg*/mL [9].

### Detection of cell viability

The viability of peritoneal macrophages treated with LPS, LPS + IFN-β, or LPS + IFN-γ were determined by trypan blue dye exclusion or a quantitative colorimetric assay with 3-(4, 5)-dimethylthiozol-2,5-diphenyl-tetrazolium bromide (MTT) as described previously [13].

### Measurement of TNF-α and iNOS gene expression levels

Thioglycolate-elicited peritoneal macrophages were prepared from 8-week-old and 46-week-old C57BL/6 mice after feeding control diet, (CD), quercetin (100 pmm), δ-tocotrienol (100 ppm), or dexamethasone (10 ppm) supplemented diets to 4-week-old and 42-week-old C57BL/6 male mice for 4 weeks. The macrophages (1 × 10^7^/well in 100 μL) of both groups were adhered for 2 h in the wells from control diet (CD), quercetin, δ-tocotrienol, or dexamethasone treatments. After 2 h all the wells were challenged with LPS (10 *ng*/well; 400 μL), and incubated at room temperature for 4 h, then macrophages were harvested, and total cellular RNA was extracted from each pellet with RNeasy mini kit (QIAGEN Sciences; Germantown, MD, USA) according to the manufacturer's instructions. Subsequent gene analyses for TNF-α and iNOS were performed after conversion of purified RNA to DNA [14].

The cDNA for each treatment was amplified and analyzed by real-time polymerase chain reaction (RT-PCR) to quantitate gene expression of TNF-α and iNOS by using 1-step RT-PCR kit (Qiagen, Chatsworth, CA, USA) according to the manufacturer's instructions [14]. The viability of peritoneal macrophages treated with LPS or LPS + various compounds were also determined by trypan blue dye exclusion or a quantitative colorimetric assay with 3-(4,5)-dimethylthiozol-2,5-diphenyl-tetrazolium bromide (MTT) as described previously [13].

### Microarray analyses of RNA after treatment with LPS plus control diet, quercetin, δ-tocotrienol or dexamethasone of peritoneal macrophages from 8-week-old C57BL/6 male mice

#### RNA isolation

Thioglycolate-elicited peritoneal macrophages from C57BL/6 mice (8 week-old) were prepared. The macrophages of 8-week-old were adhered to the bottom of 12 well plates (1 × 10^7 ^cells/well in 1.0 mL medium) for 2 h in the wells, then cells (macrophages) were washed with medium three times. The cells were pretreated with medium, quercetin (40 μM), δ-tocotrienol (20 μM) or dexamethasone (10 μM) for 1 h at room temperature, followed by LPS 10 *ng/*well treatment for 4 h. After 4 h, total cellular RNA was isolated from treated and untreated cells (quercetin, δ-tocotrienol, and dexamethasone) by using TRIzol reagent (Life Technologies, Gaithersburg, MD, USA), which was further purified using an affinity resin column (RNeasy mini kit; Qiagen, Chatsworth, CA, USA). Total RNA thus isolated was converted to cDNA by use of the Superscript cDNA synthesis kit (GIBCO-BRL, Gaithersburg, MD, USA). Double-stranded cDNA was then purified by phase lock gel (Eppendorf, Westbury, NY, USA) with phenol-chloroform extraction [14].

#### Sample preparation, fragmentation, array hybridization, and scanning

The purified cDNA was used as a template for the *in vitro *transcription reaction for the synthesis of biotinylated cRNA with the use of RNA transcript labeling reagent (Affymetrix). This labeled cRNA was fragmented and hybridized onto the Mouse Genome 430 2.0 array as described [15]. Briefly, appropriate amounts of fragmented cRNA and control oligonucleotide B2 were added along with control cRNA (BioB, BioC, BioD), herring sperm DNA, and BSA to the hybridization buffer. The hybridization mixture was heated at 99°C for 5 min followed by incubation at 45°C for 5 min before the sample was injected into the microarray. Then, the hybridization was carried out at 45°C for 16 h with mixing on a rotisserie at 60 rpm. After hybridization, the solutions were removed, and the arrays were washed and stained with streptavidin/phycoerythrin (Molecular Probes, Eugene, OR). After washes, probe arrays were scanned using the Affymetrix Gene-Chip confocal scanner at the Mayo Clinic (Rochester, MN), as described previously [15].

#### Data analyses

Gene-Chip 3.0 (Affymetrix) was used to scan and quantitatively analyze the scanned image. Once the probe array had been scanned, Gene-Chip software automatically calculated intensity values for each probe cell and made a presence or absence call for each mRNA. Algorithms in the software used probe cell intensities to calculate an average intensity for each set of probe pairs representing a gene that directly correlated with the amount of mRNA. Gene expression data were first imported in the genespring program (Agilent, Palo Alto, CA, USA), and the numbers were corrected for differences in the arrays. ALL numbers were corrected for differences in the arrays and have been scaled to a factor of 500 during the data extraction process. LPS/medium, LPS + inhibitor/medium, or inhibitor/medium, log ratio values were normalized to a scale of 0 (instead of 1, which shows decimals) and expression values of upregulated genes showed positive numbers, whereas the downregulated genes showed negative numbers. These ratios were imported into the Ingenuity Pathways Analysis software (Ingenuity Systems, Mountain View, CA, USA). Genes identified as statistically different in terms of activation from control cells were analyzed and mapped into different pathways.

### Statistical Analysis

The overall analyses identified significant main effects and interaction for all markers of various compounds compared to control group and within the groups were evaluated. Stat-View software (version 4.01) was used for the analyses of treatment-mediated effects as compared to control group (1992; Abacus Concepts, Berkeley, CA, USA). Treatment-mediated differences in various inflammatory markers variables were identified using a two-way analysis of variance (ANOVA). When the F test indicated a significant effect, the differences between the means were analyzed by a Fisher's Protected Least Significance Difference (LSD) test. Data were reported as means ± SD in text and Tables. The statistical significance level was set at 5% (***P ***< 0.05).

## Results

At the start of the experiment, four groups of 4-week-old and 42-week-old mice were fed control diet alone or three experimental diets (control diet supplemented with quercetin. δ-tocotrienol, or dexamethasone) for 4 weeks. At the end of the feeding period, the results of 8-week-old and 46-week-old mice were compared, described and discussed throughout this paper.

### Effects of dietary supplementation of quercetin, δ-tocotrienol, or dexamethasone on body weight gain

The first question that we wanted to answer was whether supplementation of commercial diet (control diet) with quercetin, δ-tocotrienol, or dexamethasone would affect body weight gains in either age group of male C57BL/6 mice. Young (4-week-old) and senescent (42-week-old) C57BL/6 male mice were fed control diet or control diet supplemented with quercetin (100 ppm), δ-tocotrienol (100 ppm), or dexamethasone (10 ppm) for 4 weeks, and body weight gains were determined after this 4 week feeding period. As shown in Table 1, when fed control diet alone, the weight of 8-week-old and 46-week-old mice increased by an average of 29% and 23%, respectively, over the 4 week period of feeding. Mice whose diet was supplemented with dexamethasone, gained significantly less weight than mice fed control diet alone (10% and 6% for 8-week-old and 46-week-old mice, respectively) (Table 1). These results confirm our earlier findings in which feeding a diet supplemented with dexamethasone to chickens significantly reduced weight gain (> 75% reduction; ***P ***< 0.05) compared to control diet [5]. Mice whose diets were supplemented with quercetin or δ-tocotrienol had weight gains that did not differ significantly from mice fed control diet. Overall consumption of food by mice in each group was similar, ranging between 245 and 256 g over the four week period of feeding, so dietary supplementation did not appear to have a substantial effect on food intake. Thus, the results of these studies demonstrate that diet supplementation with either quercetin or δ-tocotrienol does not have a significant impact on body weight gain (Table 1).

**Table 1:** Effects of various compounds on weight gain of 8-week-old and 46-week-old C57BL/6 male mice after feeding for four weeks^1^

NO		Weight gain in gm (8-week-old)		Weight gain in gm (46-week-old)	
	**C57BL/6 male mice^2^**	**Pre-Feeding**	**Post-Feeding**	**Pre-Feeding**	**Post-Feeding**

					
1	Control Diet (CD)	21.43 ± 2.25^a ^(100)^3^	27.54 ± 2.56^b ^(129)^3^	30.32 ± 2.63^a ^(100)^3^	37.31 ± 3.46^b ^(123)^3^
2	CD + Quercetin (100 ppm)	22.72 ± 2.42^a ^(100)	27.92 ± 2.37^b ^(123)	28.47 ± 2.52^a ^(100)	36.62 ± 3.76^b ^(129)
3	CDA + δ-Tocotrienol (100 ppm)	20.25 ± 2.26^a ^(100)	26.42 ± 2.51^b ^(130)	30.33 ± 2.64^a ^(100)	37.32 ± 3.38^b ^(123)
4	CD + Dexamathasone (10 ppm)	21.47 ± 2.62^a ^(100)	23.53 ± 2.41^a ^(110)	31.58 ± 3.22^a ^(100)	33.61 ± 3.21^a ^(106)

^1^Each compound (200 mg or 20 mg) was dissolved in ethanol (100 mL) and mixed with powder commercial diet (2 kg).

^2^Data are means ± SD, *n *= 3 mice per group.

^3^Percentage of gain in weight in each group compared to respective control group are in parenthesis.

^a-b^Values in a row not sharing a common superscript letter are significantly different at *P *< 0.05.

### Effects of dietary supplementation of quercetin, δ-tocotrienol, or dexamethasone on histological analyses of liver samples

Mice fed diets supplemented with dexamethasone had reduced weight gains compared to mice fed δ-tocotrienol or quercetin supplemented diets, or control diets (Table 1). Despite these differences, mice fed diets supplemented with dexamethasone were active, and there were no obvious abnormalities in physical appearance or behavior. As a preliminary assessment of potential toxicities attributable to the diet supplements, we conducted a histological analysis of liver samples from mice fed control and supplemented diets. Semi-quantitative histological analysis of these liver samples was carried out as described previously [11]. Mean scores (MS) were assigned for each variable for each sample, with scores ranging from 0 (normal appearance) to 40 (very severe impact)

**Figure 2:**
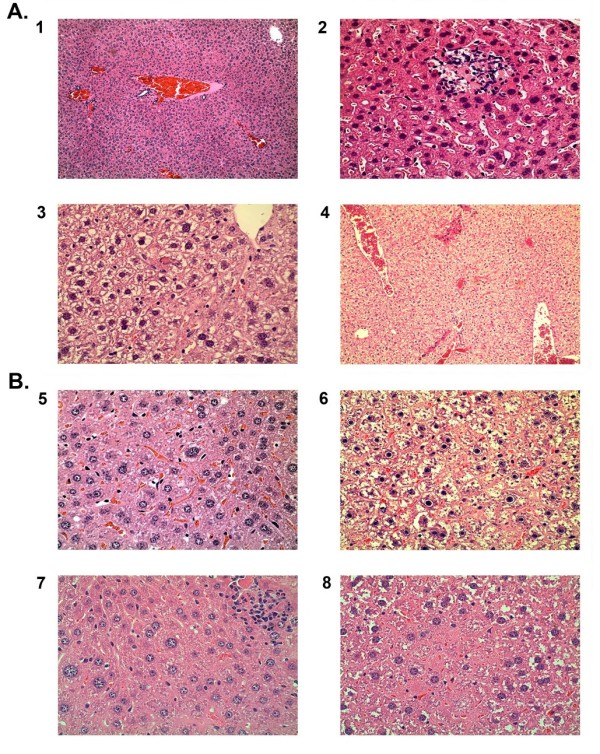
**Effects of dietary supplementation with various compounds on histological analyses of livers of 8-week-old and 46-week-old C57BL/6 male mice**: The scan 1-8 shows the histological evaluation of representative liver sections from mice fed commercial diets with and without quercetin (100 ppm), δ-tocotrienol (100 ppm), and dexamethasone (10 ppm) to 4-week-old and 42-week-old male C57BL/6 mice for 4 weeks. The livers of each mouse was processed as described in experimental section. A = 8-week-old mice: 1. control; 2. quercetin; 3. δ-tocotrienol; 4. dexamethasone; B = 46-week-old mice, 5. control; 6. quercetin; 7. δ-tocotrienol; 8. dexamethasone.

Analysis of livers from the control groups of 8-week-old and 46-week-old mice showed peliosis (MS = 15), lymphoplasmacytic clusters (MS = 15), nuclear dysplasia (MS = 15) and nuclear retreat (MS = 15) (Table 2; Figure 2). Small or large lymphoplasmacytic clusters were found in liver samples of quercetin (MS = 20) and δ-tocotrienol (MS = 20) fed mice. Liver samples of 8-week-old dexamethasone fed mice showed several pathological alterations, such as the presence of Councilman body (MS = 40), microvesicular steatosis (MS = 20), nuclear dysplasia (MS = 20), and nuclear retraction globules (MS = 20). Liver samples of 46-week-old dexamethasone fed mice showed large lymphoplasmacytic clusters (MS = 20), Councilman body (MS = 15), nuclear retreat (MS = 15) and dysplasia (MS = 15) (Figure 2). Based on the presence of a large number of councilman body (MS = 40), nuclear retreat (MS = 20), and other abnormalities in liver samples of dexamethasone fed mice, it appears that greater toxicity is associated with diet supplementation with dexamethasone compared to diet supplementation with either quercetin or δ-tocotrienol (Table 2, Figure 2).

**Table 2:** Effects of various compounds after feeding C57BL/6 of 8-week-old and46-week-old C57BL/6 male mice on histological analyses of liver samples^1^

#	SET I (8-week-old)	Summary
		
1	Control diet (CD)	Peliosis, nuclear dysplasia and nuclear retreat.
		
2	CD + Quercetin	Small & large lymphoplasmacytic clusters.
		
3	CD + δ-Tocotrienol	Large lymphoplasmacytic (LP) cluster, Councilman body.
		
4	CD + Dexamethasone	Councilman body, nuclear retreat and dysplasia, microvesicular steatosis, large lymphoplasmacytic (LP) cluster, closed sinusoids, cytoplasmic condensation.

	**SET II (46-week-old)**	**Summary**

		
5	Control diet (CD)	LP clusters, nuclear Retreat and dysplasia, microvesicular steatosis.
		
6	CD + Quercetin	Coucilman body, nuclear dysplasia and nuclear retreat.
		
7	CD + δ-Tocotrienol	Small & large Lymphoplasmacytic clusters.
		
8	CD + Dexamethasone	Small LP cluster, Councilman body, nuclear Retreat and dysplasia cytoplasmic condensation.

^1^Young (4-week-old) and senescent (42-week-old) C57BL/6 male mice were fed control diet or control diet supplemented with quercetin (100 ppm), δ-tocotrienol (100 ppm), or dexamethasone (10 ppm) for 4 weeks. After 4 weeks of feeding, thioglycolate-elicited peritoneal macrophages were prepared [9,10], then mice were sacrificed, and liver samples were removed, stored in 10% formalin at -70°C until histological analyses were carried out. After the liver tissues were fixed in formalin, they were embedded in the paraffin, and cut in the sagital plane. The sections were stained by hematoxylin and eosin, and examined under light microscopy. A semi-quantitative evaluation of histological analyses were carried out as described previously [11]. Mean scores (MS) were assigned to each sample, scored ranged from 0 (normal appearance) to 40 (very severe impact). The means of the assigned values for each group were reported

### Effects of dietary supplementation of quercetin, δ-tocotrienol, or dexamethasone on nitric oxide (NO) production by LPS-stimulated macrophages

We have previously reported that peritoneal macrophages derived from C57BL/6 female mice treated *in vitro *with quercetin, δ-tocotrienol, or dexamethasone have depressed NO responses to LPS-stimulation [3]. As enhanced NO production associated with ageing has been implicated in a variety of age-related diseases [1], we were extremely interested in determining whether macrophages derived from mice whose diets were supplemented with quercetin, δ-tocotrienol, or dexamethasone would have similarly depressed NO responses to LPS-stimulation. Mice were fed control diet or diets supplemented as described above. After 4 weeks, mice were sacrificed, and thioglycolate-elicited peritoneal macrophages were collected and challenged with LPS (10 *ng *or 100 *ng*/well) for 18 h, and NO was measured in cell culture supernatants.

**Figure 3:**
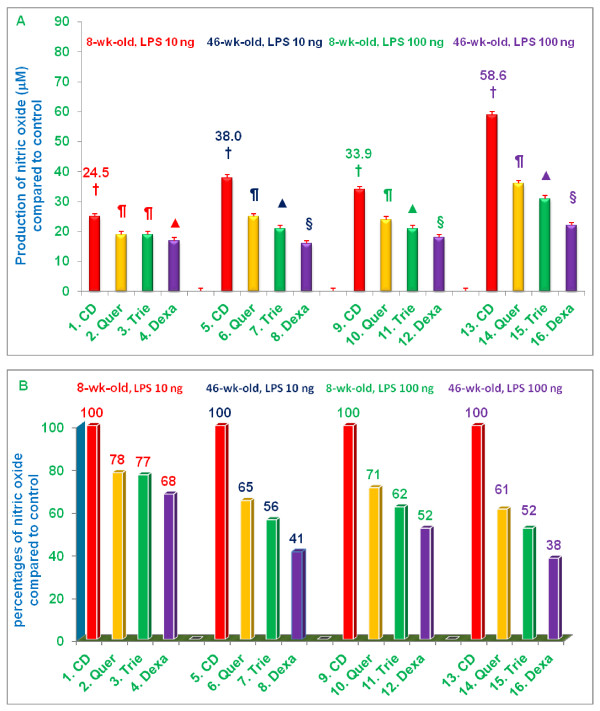
**Effects of dietary supplementation with various compounds on the production of nitric oxide (μM) by LPS-stimulated thioglycolate-elicited peritoneal macrophages**. 4-week-old and 42-week-old C57BL/6 male mice were fed control diet or control diets supplemented with quercetin, δ-tocotrienol or dexamethasone for 4 week. After this, thioglycolate-elicited peritoneal macrophages were collected, adhered to the bottom of 12 well plates (1×10^7 ^cells/well in 1.0 mL medium) for 4 h, then washed with medium three times. Cells were then treated with LPS (10 *ng*/well or 100 *ng*/well) for 18 h at room temperature, the mixtures were centrifuged at 2000 rpm for 20 min, and supernatants were collected, and stored at -70°C for subsequent analysis of NO using Griess reagent [12]. Cell viability exceeded 95% in all experiments [13]. Data represent means ± SD; *n *= 3 mice per group with triplicate analysis of each sample. Values in columns with different symbols differ at ***P ***< 0.05. Treatments 1 - 16 correspond to: 1 - 4 = 8-week-old or 5 - 8 = 46-week-old mice treated with 10 *ng*/well LPS; 9 - 12 = 8-week-old or 13 - 16 = 46-week-old mice treated with 100 *ng*/well LPS. CD = control diet; Quer = quercetin; Trie = δ-tocotrienol; Dexa = dexamethasone.

First, we wanted to determine whether age affected the ability of mice to respond to LPS as measured by NO production. As shown in Figure 3A and 3B, 46-week-old mice had a significantly more robust NO response to LPS-stimulation than 8-week-old mice as shown in Figure 3A (columns 1 vs 5 [24.58 vs 38.04 μM] for 10 *ng *LPS, and 9 vs 13 [33.90 vs 58.55 μM] for 100 *ng *LPS). When stimulated with 10 *ng *LPS, NO production by macrophages from 46-week-old mice was 55% (***P ***< 0.05) higher than that of 8-week-old mice fed control diet. With 100 *ng *LPS, NO production was 73% (***P ***< 0.02) higher in 46-week-old vs. 8-week-old mice fed control diet (Figure 3A). These results strongly support and confirm earlier observations that senescence enhances the NO responses of murine macrophages to LPS-stimulation [4].

Next, we were interested in determining the extent to which diet supplementation with quercetin, δ-tocotrienol, or dexamethasone might suppress NO production in response to LPS. Compared to mice fed control diet, macrophages from mice fed control diet supplemented with dexamethasone produced significantly less NO **(*P ***< 0.02) when stimulated with either 10 *ng *(32% and 59% reduction in NO production by 8-week-old and 46-week-old mice, respectively), or 100 *ng *LPS (48% and 62% reduction in NO production, respectively) (Figure 3B). Although the suppressive effect of diet supplementation with dexamethasone on NO production was observed in both young and senescent mice, it was more pronounced in senescent compared to young mice.

Diet supplementation with quercetin and δ-tocotrienol produced results comparable to dexamethasone (Figure 3A,B). NO production by macrophages stimulated with either 10 *ng *or 100 *ng *LPS was significantly **(*P ***< 0.02) reduced in macrophages derived from mice fed control diet plus quercetin or control diet plus δ-tocotrienol compared to those derived from mice fed control diet alone. Again, the suppressive effects of diet supplementation on NO production was observed in both young and senescent mice, but was more pronounced in senescent compared to young mice (Figure 3B). Thus, diet supplementation with quercetin, δ-tocotrienol, or dexamethasone significantly reduced the ability of LPS-stimulated macrophages to produce NO, and these suppressive effects were more pronounced in senescent, compared to young, mice (Figure 3B).

### Effects of dietary supplementation of quercetin, δ-tocotrienol, or dexamethasone on nitric oxide (NO) production by macrophages stimulated with both LPS + interferon-β (IFN-β)

Endogenous β-interferon (IFN-β) is an essential cofactor in LPS-induced NO production by macrophages, and exogenous IFN-β has been shown to enhance NO production by LPS-stimulated macrophages from both young and senescent mice [4]. Thus, the experiments described above were essentially duplicated, except that macrophages were stimulated with either LPS alone (10 *ng*) or LPS (10 *ng*) plus IFN-β (50 U/well) to determine the effects of diet supplementation with quercetin or δ-tocotrienol on nitric oxide (NO) production by macrophages stimulated with both LPS and IFN-β.

**Figure 4:**
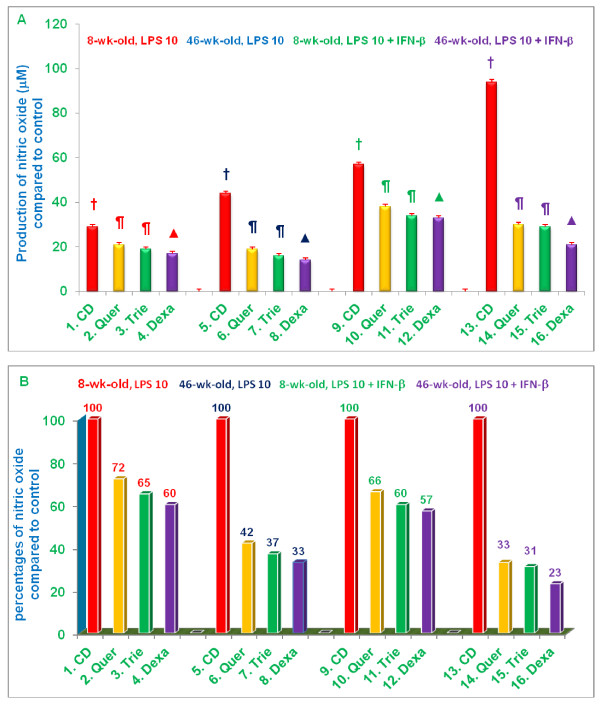
**Effects of dietary supplementation with quercetin, δ-tocotrienol, or dexamethasone on the production of nitric oxide by thioglycolate-elicited peritoneal macrophages stimulated with LPS-plus IFN-β</ττ>**. 4-week-old and 42-week-old C57BL/6 male mice were fed control diet (CD) or control diet supplemented with quercetin, δ-tocotrienol or dexamethasone for 4 week. After this, thioglycolate-elicited peritoneal macrophages were collected, adhered to the bottom of 12 well plates (1×10^7 ^cells/well in 1.0 mL medium) for 4 h, then washed with medium three times. Cells were treated with LPS (10 *ng*/well) or LPS (10 *ng *+ IFN-β 50 U/well) for 18 h at room temperature, mixtures were centrifuged at 2000 rpm for 20 min. The supernatants were collected, and were assayed for production of NO by using Griess reagent [12]. Cell viability exceeded 95% in all experiments [13]. Data represent means ± SD; *n *= 3 mice per group, with triplicate analysis of each sample. Values in columns with different symbols differ at ***P ***< 0.05. Treatments 1 - 16 correspond to: 1 - 4 = 8-week-old mice or 5 - 8 = 46-week-old mice treated with 10 *ng*/well LPS; 9 - 12 = 8-week-old mice or 13 - 16 = 46-week-old mice treated with 10 *n*g LPS plus + 50 U/well IFN-β. CD = control diet; Quer = quercetin; Trie = δ-tocotrienol; Dexa = dexamethasone.

Data presented in Figure 4A,B are consistent with those presented in Figure 3. When fed a control diet, 46-week-old mice had a significantly more robust NO response (43.92 μM; column 5, Figure 4A) to LPS-stimulation than 8-week-old mice (28.84 μM; column 1, Figure 4A). As expected (Figure 4A), macrophages derived from both young and senescent mice fed control diet, produced significantly (***P ***< 0.02) more NO when stimulated with both LPS and IFN-β [columns 9 (56.81 μM) and 13 (93.61 μM) for young and senescent mice, respectively] compared to stimulation with LPS alone [column 1 (28.84 μM) and column 5 (43.92 μM) for young and senescent mice, respectively]. The production of NO by macrophages from senescent mice stimulated with both LPS and IFN-β was particularly robust. Diet supplementation with quercetin, tocotrienol, or dexamethasone significantly suppressed NO production by macrophages stimulated with either LPS alone, or LPS plus IFN-β. This suppression was observed with macrophages derived from both young and senescent mice, but the extent of suppression (67-77%; columns 14-16) was greatest in LPS plus IFN-β stimulated macrophages derived from senescent mice (Figure 4A,B).

### Effects of dietary supplementation of quercetin, δ-tocotrienol, or dexamethasone on nitric oxide (NO) production by macrophages stimulated with both LPS + interferon-γ (IFN-γ)

Interferon-γ (IFN-γ) is a potent macrophage activator that synergizes with a variety of other macrophage activators to enhance NO production, and exogenous IFN-γ has been shown to enhance NO production by LPS stimulated macrophages from both young and senescent mice [4]. Thus, the experiments described above were essentially duplicated, except that macrophages were stimulated with either LPS alone (10 *ng*) or LPS (10 *ng*) plus IFN-γ (50 U/well) to determine the effects of diet supplementation with quercetin, or δ-tocotrienol on nitric oxide (NO) induction by macrophages stimulated with both LPS and IFN-γ.

**Figure 5:**
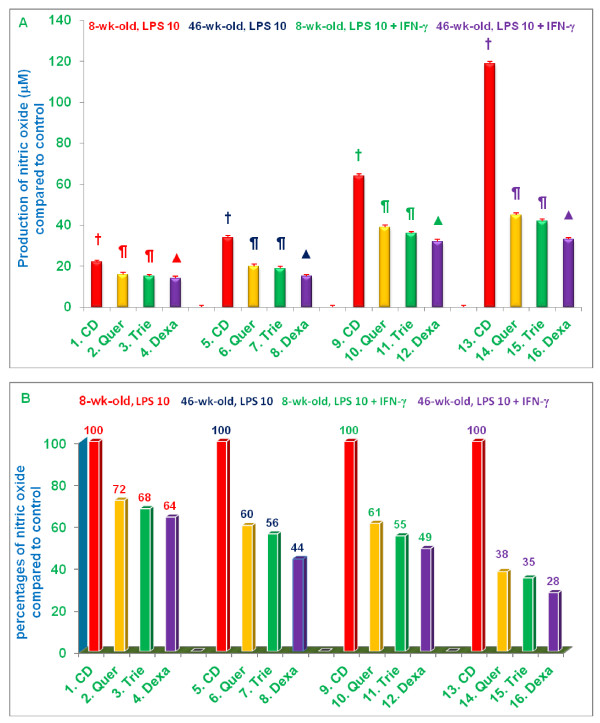
**Effects of dietary supplementation with quercetin, δ-tocotrienol, or dexamethasone on the production of nitric oxide by thioglycolate-elicited peritoneal macrophages stimulated with LPS-plus IFN-γ</ττ>**. 4-week-old and 42-week-old C57BL/6 male mice were fed control diet (CD) or control diet supplemented with quercetin, δ-tocotrienol or dexamethasone for 4 week. After this, thioglycolate-elicited peritoneal macrophages were collected, adhered to the bottom of 12 well plates (1×10^7 ^cells/well in 1.0 mL medium) for 4 h, then washed with medium three times. Cells were treated with LPS (10 *ng*/well) or LPS 10 *ng *+ IFN-γ 50 U/well) for 18 h at room temperature, mixtures were centrifuged at 2000 rpm for 20 min. The supernatants were collected and assayed for production of NO by using Griess reagents [12]. Cell viability exceeded 95% in all experiments [13]. Data represent means ± SD; *n *= 3 mice per group, with triplicate analysis of each sample. Values in columns with different symbols differ at ***P ***< 0.05. Treatments 1 - 16 correspond to: 1 - 4 = 8-week-old mice or 5 - 8 = 46-week-old mice treated with 10 *ng*/well LPS; 9 - 12 = 8-week-old mice or 13 - 16 = 46-week-old mice treated with 10 *n*g LPS plus + 50 U/well IFN-γ. CD = control diet; Quer = quercetin; Trie = δ-tocotrienol; Dexa = dexamethasone.

Data presented in Figure 5A,B are consistent with those presented in Figures 3 and 4. When fed a control diet, 46-week-old mice had a significantly (***P ***< 0.05) more robust NO response (33.78 μM; Figure 5A, column 5) to LPS-stimulation than 8-week-old mice (22.29 μM; Figure 5A, column 1). As expected (Figure 5A), macrophages derived from both young and senescent mice fed control diet, produced significantly (***P ***< 0.02) more NO when stimulated with both LPS and IFN-β [columns 9 (64.18 μM) and 13 (118.72 μM) for young and senescent mice, respectively] compared to stimulation with LPS alone [columns 1 (22.29 μM) and 5 (33.78 μM) for young and senescent mice, respectively]. NO production by macrophages from senescent mice stimulated with both LPS and IFN-γ was particularly robust. Diet supplementation with dexamethasone, quercetin, or δ-tocotrienol significantly suppressed NO production by macrophages stimulated with either LPS alone, or LPS plus IFN-γ. This suppression was observed with macrophages derived from both young and senescent mice, but the extent of suppression (62-72%; columns 14-16) was greatest in LPS plus IFN-γ stimulated macrophages derived from senescent mice (Figure 5A,B)

### Effects of dietary supplementation of quercetin, δ-tocotrienol, or dexamethasone on TNF-α secretion by LPS-stimulated macrophages

**Figure 6:**
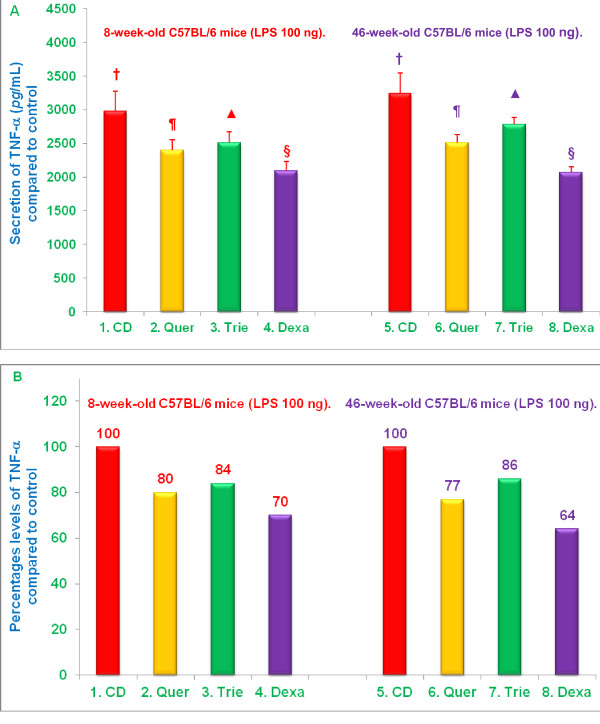
**Effects of dietary supplementation with various compounds on the secretion of TNF-α by thioglycolate-elicited peritoneal macrophages stimulated with LPS**. 4-week-old and 42-week-old C57BL/6 male mice were fed control diet or control diet supplemented with quercetin, δ-tocotrienol or dexamethasone for 4 week. After this, thioglycolate-elicited peritoneal macrophages were collected, adhered to the bottom of 12 well plates (1×10^7 ^cells/well in 1.0 mL medium) for 4 h, then washed with medium three times. Then cells were treated with LPS (100 *ng*/well), and incubated at 37°C in 5% CO2 for 4 h [9]. Supernatants were used to carry out TNF-α assays by ELISA. Data are means ± SD; *n *= 3 mice per group, with triplicate analysis of each sample. Values in columns with different symbols differ at ***P ***< 0.05. The treatments 1 - 8 correspond to: 1 - 4 = 8-week-old mice or 5 - 8 = 46-week-old mice treated with 100 *ng *LPS. CD = control diet; Quer = quercetin; Trie = δ-tocotrienol; Dexa = dexamethasone.

TNF-α is among the earliest and most important pro-inflammatory cytokines produced in response to a variety of inflammatory stimuli. We have previously demonstrated that proteasome inhibitors can suppress TNF-α secretion by LPS-stimulated macrophages *in vitro*, so we opted to determine the extent to which diet supplementation with quercetin, δ-tocotrienol, or dexamethasone might suppress TNF-α secretion in response to LPS [4]. Mice were fed the same control and supplemented diets described in the previous sections. After 4 weeks, thioglycolate-elicited peritoneal macrophages were collected and challenged with 100 *ng*/well LPS for 4 h, and TNF-α was measured in cell culture supernatants. In contrast to the results with NO, age did not have a significant effect on TNF-α secretion by LPS-stimulated macrophages; TNF-α levels were comparable in supernatants of LPS-stimulated peritoneal macrophages from 8-week-old (2,980 *pg*/mL) and 46-week-old (3,241 *pg*/mL) mice fed control diet (Figure 6A,B). LPS-stimulated macrophages from both young and senescent mice fed control diet supplemented with quercetin, δ-tocotrienol, or dexamathasone, however, produced significantly less (***P ***< 0.05) TNF-α than macrophages from mice fed control diet (Figure 6A,B). Control diet supplementation with dexamathasone, quercetin, or δ-tocotrienol suppressed TNF-α secretion by 30%, 20%, and 16% respectively, in 8-week-old mice, and by 36%, 23%, and 14%, respectively in 46-week-old mice. The extent of the reduction in TNF-α secretion was comparable in both age groups (Figure 6A,B). Thus, diet supplementation with quercetin, δ-tocotrienol, or dexamethasone significantly reduces the ability of LPS-stimulated macrophages to produce TNF-α (Figure 6A,B).

### Effects of diet supplementation with quercetin, δ-tocotrienol, or dexamethasone on expression levels of TNF-α and iNOS genes in LPS-stimulated macrophages

The results presented above collectively demonstrate that increasing age enhances NO production, and diet supplementation with quercetin, δ-tocotrienol, or dexamethasone suppresses production of NO and TNF-α by LPS-stimulated macrophages from both young and senescent mice. The next series of experiments were designed to determine whether the changes observed in NO and TNF-α production attributable to age or diet supplementation were associated with comparable changes in mRNA expression levels for the TNF-α and iNOS genes.

**Figure 7:**
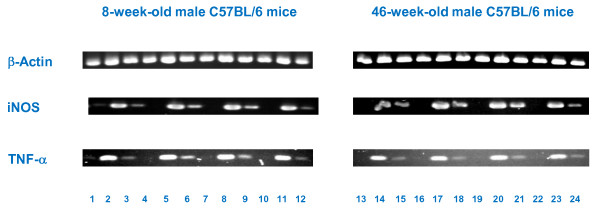
**Effects of dietary supplementation of various compounds on the gene expression of iNOS and TNF-α in LPS-stimulated thioglycolate-elicited peritoneal macrophages derived from 8-week-old and 46-week-old C57BL/6 male mice after feeding for 4 weeks**. 4-week-old and 42-week-old C57BL/6 male mice were fed control diet or control diet supplemented with quercetin, δ-tocotrienol or dexamethasone for 4 week. After this, thioglycolate-elicited peritoneal macrophages were collected, adhered to the bottom of 12 well plates (1×10^7 ^cells/well in 1.0 mL medium) for 4 h, then washed with medium three times. The cells were treated with LPS (10.0 *ng*/well) for 4 h, and the purified RNA of each mouse was subjected to RT-PCR analyses, as described in the experimental section. The top rows in Figure 7, correspond to β-Actin, middle rows, iNOS and bottom rows, TNF-α (8-week-old mice, 1-12, and 46-week-old mice, 13 - 24). ***8-week-old mice***, f**or iNOS (middle row, 1-12), and TNF-α (bottom row, 1-12)**: 1. Medium + macrophages = cells, 2. Cells + LPS 10 *ng*/well, 3. Cells + LPS 1 *ng*/well; 4. Medium + macrophages = cells, 5. Cells + LPS 10 *ng*/well + quercetin, 6. Cells + LPS 1 *ng*/well + quercetin; 7. Medium + macrophages = cells, 8. Cells + LPS 10 *ng*/well + δ-tocotrienol, 9. Cells + LPS 1 *ng*/well + δ-tocotrienol; 10. Medium + macrophages = cells, 11. Cells + LPS 10 *ng*/well + dexamethasone 12. Cells + LPS 1 *ng*/well + dexamethasone. ***46-week-old mice***, f**or iNOS (second row, 13-24), and TNF-α (third row, 13-24)**: 13. Medium + macrophages = cells, 14. Cells + LPS 10 *ng*/well, 15. Cells + LPS 1 *ng*/well; 16. Medium + macrophages = cells, 17. Cells + LPS 10 *ng*/well + quercetin, 18. Cells + LPS 1 *ng*/well + quercetin; 19. Medium + macrophages = cells, 20. Cells + LPS 10 *ng*/well + δ-tocotrienol, 21. Cells + LPS 1 *ng*/well + δ-tocotrienol; 22. Medium + macrophages = cells, 23. Cells + LPS 10 *ng*/well + dexamethasone 24. Cells + LPS 1 *ng*/well + dexamethasone.

Young (4-week-old) and senescent (42-week-old) C57BL/6 male mice were fed control diet or control diets supplemented with quercetin (100 ppm), δ-tocotrienol (100 ppm), or dexamethasone (10 ppm) for 4 weeks. After 4 weeks, 8-week-old and 46-week-old mice were sacrificed, and thioglycolate-elicited peritoneal macrophages were prepared, and challenged with LPS (10 *ng*/well) for 4 h. Cells were harvested, total cellular RNA extracted, and targeted genes were amplified and analyzed by RT-PCR to determine relative expression levels. As shown in Table 3 and Figure 7, mRNA levels for TNF-α were roughly comparable in LPS stimulated macrophages from 8-week-old and 46-week-old mice fed control diet. In contrast, mRNA levels for iNOS were substantially higher in LPS- stimulated macrophages from 46-week-old compared to 8-week-old mice fed control diet.

**Table 3:** Effects of various compounds on the gene expression of TNF-α and iNOS in LPS-stimulated thioglycolate-elicited peritoneal macrophages derived from 8-week-old and 46-week-old C57BL/6 male mice after feeding for 4 weeks^1^.

8-week-old vs 46-week-old		RT-PCR (8-wk-old) data^2^		RT-PCR (46-wk-old) data^2^	
**NO**		**Gene Expression**		**Gene Expression**	

	**C57BL/6 Male Mice**	**TNF-α**	**iNOS**	**TNF-α**	**iNOS**

					
1	Media + Macrophages (MM)	0	0	0	0
2	MM + LPS, 10.0 *ng*/mL (A)	0.35 (100)*^3^	0.41 (100)^3^	0.38 (100)^3^	0.69 (100)^3^
3	A + Quercetin (100 ppm)	0.26 (74)	0.29 (71)	0.29 (76)	0.47 (68)
4	A + δ-Tocotrienol (100 ppm)	0.24 (69)	0.22 (54)	0.20 (53)	0.28 (41)
5	A + Dexamethasone (10 ppm)	0.20 (57)	0.15 (37)	0.27 (71)	0.20 (29)
					

					
	
	*Control groups	**8-week-old vs**	**46-week-old**		
	
					
	**TNF-α**	**0.35 (100)**	**0.38 (109)**		
					
	**iNOS**	**0.41 (100)**	**0.69 (168)**		
					

^1^The assay mixture (A; contains 0.2% dimethyl sulfoxide = DMSO) was treated with LPS (10.0 *ng*/well) for 4 h.

^2^Total RNA was extracted using RNAeasy mini Kit.

^3^Percentage of control ratio, based on MM + LPS 10.0 *ng*/well.

LPS-stimulated macrophages from 8-week-old and 46-week-old mice whose diets were supplemented with quercetin, δ-tocotrienol, or dexamethasone had reduced levels of mRNA for both the iNOS and TNF-α genes, compared to macrophages from mice fed control diet. Thus, results at the gene expression level for iNOS and TNF-α were entirely consistent with results of NO production and TNF-α secretion by LPS-stimulated macrophages. iNOS mRNA levels and NO production were higher in LPS-stimulated macrophages from senescent compared to young mice. In contrast, TNF-α RNA and secretion of TNF-α protein by LPS-stimulated macrophages did not appear to be affected by age. Diet supplementation with dexamethasone, quercetin, or δ-tocotrienol for 4 weeks resulted in decreased mRNA expression levels and decreased secretion of TNF-α protein and NO by LPS-stimulated macrophages from both senescent and young mice (Table 3).

### Effects of quercetin, δ-tocotrienol, or dexamethasone on gene transcription in LPS-stimulated thioglycolate-elicited peritoneal macrophages as assessed by microarray analyses

The results of experiments presented above demonstrated that production of NO and TNF-α, and transcription of iNOS and TNF-α genes, was reduced in both young and senescent mice fed diets supplemented with quercetin, δ-tocotrienol, or dexamethasone for 4 weeks. These results were further supported by microarray analyses of RNA purified from LPS-stimulated thioglycolate-elicited peritoneal macrophages of 8-week-old C57BL/6 male mice pretreated with quercetin, δ-tocotrienol, or dexamethasone for 1 h, followed by LPS 10 *ng*/treatment for 4 h. Total cellular RNA was extracted as described in the experiment section. Extracted RNA was converted to cDNA according to Affymetrix expression analysis technical manual.

The data in Tables 4, 5, and 6 represent the number of genes modulated by LPS; and LPS plus quercetin, dexamethasone and δ-tocotrienol; and quercetin, dexamethasone, and δ-tocotrienol alone. The LPS-modulated genes have been categorized with respect to functions, that is, specifically immune response, cell to cell signaling, cellular growth and proliferation, inflammatory diseases, respiratory diseases, immunological diseases, cancer, hepatic system diseases, cardiovascular system, lipid metabolism, neurological diseases, and gene expression. It is clear from these Tables that several functions are affected by treatment with LPS, and LPS plus quercetin, dexamethasone or δ-tocotrienol, or inhibitors alone. When the LPS-modulated genes were also categorized with respect to canonical pathways, such as C21-steroid hormone metabolism, Notch signaling, serotinin receptor signaling, retinol metabolism, toll-like receptor signaling, and bile acid synthesis (Table 7). Similarly, it seems clear that treatment with LPS, and LPS plus quercetin, dexamethasone or δ-tocotrienol, or inhibitors alone also affects the canonical pathways as well. The individual genes that were either up-regulated or down-regulated by the various treatments are described in Tables 8, 9, and 10.

**Table 4:** Effects of LPS, LPS + quercetin, and quercetin on the levels of gene expression, and biological functions in macrophages of8-week-old C57BL/6 male mice^1^

			Regulation		Regulation			Regulation		
**#**	**Functions**	**LPS**	**Up**	**Down**	**LPS + Quer**	**Up**	**Down**	**Quer**	**Up**	**Down**

										
1	Immune response	**182**	130	52	**124**	84	40	**39**	9	30
2	Cell to cell signaling	**124**	86	38	**136**	78	58	**41**	4	37
3	Cellular growth & proliferation	**74**	54	20	**99**	61	38	**36**	22	14
4	Inflammatory diseases	**74**	53	21	**88**	61	27	**24**	1	23
5	Respiratory diseases	**44**	34	10	**42**	31	11	**27**	4	23
6	Immunological diseases	**39**	35	4	**37**	26	11	**9**	6	3
7	Cancer	**34**	25	9	**42**	28	14	**13**	9	4
8	Hepatic system diseases	**26**	23	3	**5**	5				
9	Cardiovascular system	**18**	14	4	**44**	33	11	**10**	8	2
10	Carbohydrate Metabolism	**8**	5	3	**13**	1	12			
11	Lipid metabolism	**7**	7		**53**	36	17	**39**	24	15
12	Neurological Diseases	**6**	5	1	**34**	29	5	**4**	4	
13	Free-radical scavenging	**5**	4	1				**4**	3	1
14	Gene expression	**4**	4		**7**	6	1			

**Total**		**645**	**479**	**166**	**724**	**479**	**245**	**246**	**94**	**152**

LPS = Lipopolysaccharide; Quer = Quercetin.

^1^The data in this Table represents the expression of a number of genes modulated by various treatments; The macrophages were treated with LPS alone (10 *ng*/well), LPS (10 *ng*/well) + quercetin (40 μM), or quercetin (40 μM) alone as described in the experimental section. Total RNA was extracted from the treated cells and their gene expression profiles compared with the control (LPS), and untreated cells. The values here been corrected for differences in the arrays. The gene expression values are reported as average normalization log ratios. A data containing gene identifiers and their corresponding expression values were uploaded as an Excel spreadsheet using the template provided in the Ingenuity Pathways Analysis program. Similar conditions were used for data provided in Tables 8, 9, and 10.

**Table 5:** Effects of LPS, LPS + δ-tocotrienol, and δ-tocotrienol on the levels of gene expression, and biological functions in macrophages of 8-week-old C57BL/6 male mice^1^.

			Regulation		Regulation			Regulation		
**#**	**Functions**	**LPS**	**Up**	**Down**	**LPS + Trie**	**Up**	**Down**	**Trie**	**Up**	**Down**

										
1	Immune response	**182**	130	52	**132**	80	52	**50**	40	10
2	Cell to cell signaling	**124**	86	38	**97**	46	51	**52**	40	12
3	Cellular growth & proliferation	**74**	54	20	**55**	42	13	**32**	18	14
4	Inflammatory diseases	**74**	53	21	**9**	5	4	**5**	5	
5	Respiratory diseases	**44**	36	8				**5**	5	
6	Immunological diseases	**39**	33	6	**10**	6	4	**4**	1	3
7	Cancer	**34**	25	9	**29**	22	7	**17**	12	5
8	Hepatic system diseases	**26**	23	3	**8**	7	1	**3**	2	1
9	Cardiovascular system	**18**	14	4	**33**	27	6	**6**	4	2
10	Carbohydrate Metabolism	**8**	5	3	**14**	5	9			
11	Lipid metabolism	**7**	7		**45**	35	10	**49**	34	15
12	Neurological Diseases	**6**	5	1	**26**	24	2	**4**	4	
13	Free-radical scavenging	**5**	4	1				**4**	3	1
14	Gene expression	**4**	4		**23**	19	4	**3**	3	

**Total**		**645**	**479**	**166**	**481**	**318**	**163**	**234**	**171**	**63**

LPS = Lipopolysaccharide; Trie = δ-Tocotrienol.

^1^The data in this Table represents the expression of a number of genes modulated by various treatments; macrophages were treated with LPS alone (10 *ng*/well), LPS (10 *ng*/well) + δ-tocotrienol (20 μM), or δ-tocotrienol (20 μM) alone as described in the experimental section. Total RNA was extracted from the treated cells and their gene expression profiles compared with the control (LPS), and untreated cells. The values here have been corrected for differences in the arrays. The gene expression values are reported as average normalization log ratios. A data set containing gene identifiers and their corresponding expression values were uploaded as an Excel spreadsheet using the template provided in Ingenuity Pathways Analysis Program. Similar conditions were used for data provided in Tables 8, 9, 10.

**Table 6:** Effects of LPS, LPS + dexamethasone, and dexamethasone on levels of gene expression, and biological functions in macrophages of 8-week-old C57BL/6 male mice^1^.

			Regulation		Regulation			Regulation		
**#**	**Functions**	**LPS**	**Up**	**Down**	**LPS + Dexa**	**Up**	**Down**	**Dexa**	**Up**	**Down**

										
1	Immune response	**182**	130	52	**126**	77	49	**97**	53	44
2	Cell to cell signaling	**124**	86	38	**119**	64	55	**70**	24	46
3	Cellular growth & proliferation	**74**	54	20	**84**	38	46	**18**	2	16
4	Inflammatory diseases	**74**	53	21	**83**	58	25	**17**	11	6
5	Respiratory diseases	**44**	36	8	**28**	24	4	**17**	11	6
6	Immunological diseases	**39**	33	6	**16**	12	4			
7	Cancer	**34**	25	9	**45**	34	11			
8	Hepatic diseases	**26**	23	3	**35**	31	4			
9	Cardiovascular system	**18**	14	4	**20**	13	7	**14**	10	4
10	Carbohydrate Metabolism	**8**	5	3						
11	Lipid metabolism	**7**	7		**55**	36	19	**27**	25	2
12	Neurological Diseases	**6**	5	1	**26**	23	3			
13	Free-radical scavenging	**5**	4	1				**5**	2	3
14	Gene expression	**4**	4		**25**	21	4	**18**	16	2

**Total**		**645**	**479**	**166**	**662**	**431**	**231**	**283**	**154**	**129**

LPS = lipopolysaccharide; Dexa = Dexamethasone.

^1^The data in this Table represents the expression of a number of genes modulated by various treatments; The macrophages were treated with LPS alone (10 *ng*/well), LPS (10 *ng*/well) + dexamethasone (10 μM), or dexamethasone (10 μM) alone as described in the experimental section. Total RNA was extracted from the treated cells and their gene expression profiles compared with the control (LPS), and untreated cells. The values here been corrected for differences in the arrays. The gene expression values are reported as average normalization log ratios. A data containing gene identifiers and their corresponding expression values were uploaded as an Excel spreadsheet using the template provided in the Ingenuity Pathways Analysis program. Similar conditions were used for data provided in Tables 8, 9, and 10.

**Table 7:** Effects of LPS, or LPS + with and without quercetin (Quer), δ-tocotrienol (δ-Trie) or dexamethasone (Dexa) on canonical*l*pathways in liver samples of C57BL/6 male mice.

#	Functions	LPS	LPS + Quer	Quer	LPS + δ-Trie	δ-Trie	འ	LPS + Dexa	Dexa
									
1	C21-Steroid Hormone Metabolism	11			9	8			
2	Notch Signaling	25		19	23	20		22	
3	Serotonin Receptor Signaling	14		13	13	10		11	11
4	Tyrosine Metabolism	21	20		23	16			
5	Taurine and Hypotaurine Metabolism	7	6	6	7	6		6	
6	Retinol Metabolism	5			5	5		4	
7	Toll-like Receptor Signaling	31	24		31	30			
8	Bile Acid Synthesis	20							
9	Histidine Metabolism	16							
10	Nitric Oxide Signaling in Cardiovascular					27			
11	FGF Signaling			32					
12	GABA Receptor Signaling				24	21			22
13	Dopamine Receptor Signaling			20		19			
14	Sulfur Metabolism		5	4				5	4
15	Valine, Leucine, Isoleucine Degradation				17			6	
16	Alanine and Aspartate Metabolism		16						
17	Tryptophan Metabolism			35	11	43		39	
18	Phenylalanine, Tyrosine Biosynthesis				15				
19	Cysteine Metabolism			7	7	7			
20	Pantothenate & CoA Biosynthesis				34	30			
21	Nicotinate & Nicotinamide Metabolism		16						
22	Prostaglandin & Leukotriene Metabolism		30	26					
23	Fatty Acid Biosynthesis				5	4			4

^1^The data in this Table represents the expression of a number of genes modulated by various treatments. Macrophages were treated with LPS (10 *ng*/well), quercetin (40 μM), δ-tocotrienol (20 μM), or dexamethasone (10 μM) with and without LPS (10 ng/well) as described in the experimental section. Total RNA was extracted from the various treated cells and their gene expression profiles compared with the control (LPS), and untreated cells. The values here have been corrected for differences in the arrays. The gene expression values are reported as average normalization log ratios. A data set containing gene identifiers and their corresponding expression values were uploaded as an Excel spreadsheet using the template provided in the Ingenuity Pathway Analysis programm Various functions, their location, and gene expression values in this table represent for inhibitors only. The details of comparison for specific gene expression for each inhibitor were reported in Tables 8,9,10.

**Table 8:** Microarray analyses of RNA after treatment with LPS, LPS + quercetin or quercetin of macrophages from C57BL/6 male mice.

	**Ageing**:	Genes Up-regulated by LPS				
**#**	**Genes**	**LPS**	**LPS + Quercetin**	**Quercetin**	**Description**	**Function**

1	IL-6 (IFNβ2)	341.52	124.92	-1.03	Interleukin 6 (interferon β2)	NF-κB & IL-6 Signaling
2	CD40	258.44	28.36	1.40	CD40 antigen (TNF receptor superfamily mem-5)	Signaling
3	IL-1β	249.03	38.18	1.02	Interleukin 1β	NF-κB & IL-6 Signaling
4	IFIPT	186.05	136.24	-4.67	Interferon induced protein with tetratrocopeptide	Protein
5	TRAF1	150.74	23.09	-1.97	TNF receptor associated factor 1	Signaling
6	IL1RA	125.10	113.37	34.71	Interleukin 1 receptor antagonist	Signaling
7	CD69	105.21	13.35	-3.79	CD69 antigen (P60, early T-cell activation antigen)	Antigen
8	CCND2	101.08	28.46	-4.39	Cyclin D2	Cell cycle
9	VCAM1	95.41	25.70	2.42	Vascular cell adhesion molecule	Inflammation-linked
10	RASGRP1	90.88	13.71	-9.18	Ras guanyl releasing protein 1	Protein
11	CCL8	87.72	10.33	1.19	Chemokine (C-C motif) ligand 8	Chemokine
12	IL-12β	70.51	5.47	-4.63	Interleukin 12β	NF-κB & IL-6 Signaling
13	SOCS3	62.94	24.87	-5.22	Suppressor of cytokine signling 3	Anti-inflammation
14	TNF	54.11	47.44	-1.21	Tumor necrosis factor	NF-κB & IL-6 Signaling
15	SERPIN	46.27	10.85	-1.64	Serine or cysteine proteinase inhibitor	Inhibitor
16	ABCC	42.89	-2.02	33.56	ATP-binding cassette, subfamily C	Protein
17	ICAM1	41.21	8.51	-3.56	Intercellular adhesion molecule 1	Inflammation-linked
18	IL-1α	39.92	13.49	-1.08	Interleukin 1α	NF-kB & IL-6 Signaling
19	UBE2A	10.41	18.06	13.26	Ubiquitin conjugating enzyme E2A	Enzyme
20	CNTNA1	5.41	6.35	12.69	Contactin associated proten1	Plasma membrane
21	ATF7	5.05	7.14	13.68	Activating transcription factor 7	Transcriptor
22	LRRN6A	3.42	7.98	4.36	Leucine rich repeat neuronal 6A	Enzyme

						

	**Ageing**	**Genes Down-regulated by LPS**				

#	**Genes**	**LPS**	**LPS + Quercetin**	**Quercetin**	**Description**	**Function**

						
1	ABCD2	*-43.36*	*-48.92*	*-24.78*	ATP-binding cassette, subfamily D member 2	Protein
2	RASGRP3	-36.19	-4.43	-2.46	RAS guanyl releasing protein 3 (GRP3)	Protein
3	PTBP2	*-32.63*	*-9.00*	*-15.35*	Polypyrimidine tract binding protein 2	Protein
4	IFNGR1	-28.05	-1.65	-9.69	Interferon gamma receptor 1	Signaling
5	TNP1	-24.67	-3.56	-3.70	Transition protein 1	Protein
6	PHKA2	-22.65	-3.14	4.06	Phosphorylase kinase, alpha 2 (liver)	Enzyme
7	UBE2D2	*-22.60*	*1.95*	*-22.60*	Ubiquitin conjugating enzyme E2D2	Enzyme
8	HCAP-G	-22.08	-17.67	2.86	Chromosome condensation protein G	Protein
9	4930503816	-22.71	-6.24	-6.36	RIKEN cDNA	Enzyme
10	NFATC	-5.60	-3.01	-41.42	Nuclear factor of activated T cells cytoplasmic	Transcriptor

**Table 9:** Microarray analyses of RNA after treatment with LPS, LPS + δ-tocotrienol, or δ-tocotrienol of macrophages from C57BL/6 male mice.

		Genes Up-regulated by LPS				
**#**	**Genes**	**LPS**	**LPS + δ-Tocotrienol**	**δ-Tocotrienol**	**Description**	**Function**

						
1	IL-6 (IFNb2)	341.52	184.38	8.74	Interleukin 6 (interferon β2)	NF-κB & IL-6 Signaling
2	CD40	258.44	104.31	3.75	CD40 antigen (TNF receptor superfamily mem-5)	Signaling
3	IL-1β	249.03	126.71	1.07	Interleukin 1β	NF-κB & IL-6 Signaling
4	IFPIT	186.05	143.32	-3.77	Interferon induced protein with tetratrocopeptide	Protein
5	Tgtp	78.14	40.54	1.29	T cell specific GTPase	Enzume
6	TRAF1	150.74	89.04	4.06	TNF receptor associated factor 1	Signaling
7	IL-1RA	125.74	175.73	57.18	Interleukin-1 receptor antagonist	Signaling
8	CD69	105.21	41.59	1.10	CD69 antigen (P60, early T-cell activation antigen)	Antigen
9	CCND2	101.81	43.91	5.33	Cyclin D2	Cell cycle
10	VCAM1	95.41	63.90	-4.94	Vascular cell adhesion molecule	Inflammation-linked
11	RASGRP1	90.88	47.77	3.81	Ras guanyl releasing protein 1	Protein
12	CCL8	87.72	11.31	-1.05	Chemokine (C-C motif) ligand 8	Chemokine
13	IL-12β	70.51	34.17	-1.68	Interleukin12β	NF-κB & IL-6 Signaling
14	SOCS3	62.94	39.81	-2.92	Suppressor of cytokine signalling 3	Anti-inflammation
15	TNF	54.11	53.41	-1.98	Tumor Necrosis factor	NF-κB & IL-6 Signaling
16	SERPIN	46.27	44.43	1.05	Serine or cysteine proteinase inhibitor	Inhibitor
17	ABCC	42.89	-5.22	5.00	ATP-binding cassette, subfamily C	Protein
18	ICAM1	41.21	29.89	-1.11	Intercellular adhesion molecule 1	Inflammation-linked
19	IL-1α	39.92	32.79	1.58	Interleukin 1α	NF-κB & IL-6 Signaling
20	ATP2A2	24.00	48.36	57.91	AtPase, Ca++ transporting cardiac muscle twitch	Enzyme
21	ACTL6B	22.49	23.29	14.23	Actin-like 6B	Protein
						

		**Genes Down-regulated by LPS**				

**#**	**Genes**	**LPS**	**LPS + δ-Tocotrienol**	**δ-Tocotrienol**	**Description**	**Function**

						
1	ABCD2	-43.36	-39.75	-5.51	ATP-binding cassette, subfamily D member 2	Protein
2	RASGRP3	-36.19	-19.54	-1.07	RAS guanyl releasing protein 3 (GRP3)	Protein
3	PTBP2	-32.63	-2.64	2.20	Polypyrimidine tract binding protein 2	Protein
4	IFNGR1	-28.05	-4.72	-3.75	Interferon gamma receptor 1	Signaling
5	TNP1	-24.67	-13.88	-2.87	Transition protein 1	Protein
6	PHKA2	-22.65	-6.95	2.71	Phosphorylase kinase, alpha 2 (liver)	Enzyme
7	UBE2D2	-22.60	-17.39	-7.53	Ubiquitin conjugating enzyme E2D2	Enzyme
8	4930503B16	-22.71	-1.18	-2.11	RIKEN cDNA gene	Enzyme
9	PSMB2	-1.44	-1.91	-25.58	Proteasome (prosome macropain) subunit, β-type 2	Peotidase
10	GIMAP1	-1.205	-3.16	-20.29	GTPase, IMP family member 1	Ion Exchange
						

**Table 10:** Microarray analyses of RNA after treatment with LPS, LPS + dexamethasone (Dexa), or dexamethasone (Dexa) of macrophages from 8-week-old C57BL/6 male mice.

	Ageing		Genes Up-regulated by LPS			
**#**	**Genes**	**LPS**	**LPS + Dexa**	**Dexa**	**Description**	**function**

1	IL-6 (IFNβ2)	341.52	100.53	1.21	Interleukin 6 (interferon β2)	NF-κB & IL-6 Signaling
2	CD40	258.44	100.54	6.03	CD40 antigen (TNF receptor superfamily)	Signaling
3	IL-1β	249.03	46.52	2.09	Interleukin-1β	NF-κB & IL-6 Signaling
4	IFIPT	186.05	90.83	-6.37	Interferon Induced protein with tetrarocopeptide	Protein
5	HD	152.95	8.62	3.78	Histidine decarboxylase	Enzyme
6	TRAF1	150.74	73.45	-1.74	TNF receptor-associated factor 1	Signaling
7	USP9	134.50	105.70	76.70	Ubiquitin specific protease 9	Peptidase
8	IL1RA	125.10	74.86	40.46	Interleukin 1 receptor antagonist	Signaling
9	CD69	105.21	37.08	-1.79	CD69 Antigen (p60, early T-cell activation)	Antigen
10	VCAM1	95.405	31.684	-3.435	Vascular cell adhesion molecule	Cell cycle
11	RASGRP1	90.88	13.61	1.89	RAS guanyl releasing protein 1	Inflammation-linked
12	CCL8	87.72	5.59	1.46	Chemokine (C-C motif) ligand 8	Chemokine
13	SERPIN	46.27	3.35	-2.34	Serine or cysteine proteinase inhibitor	Inhibitor
14	ABCC	42.89	-3.86	9.67	ATP-binding cassette, subfamily C	Protein
15	ICAM1	41.21	30.85	-2.12	Intercellular adhesion molecule 1	Inflammation-linked
16	IL1α	39.92	12.01	-1.90	Interleukin 1α	NF-κB & IL-6 Signaling
17	LRRC17	31.20	24.00	9.72	Leucine rich repeat containing 17	Inhibitor
18	IL-12α	29.59	6.65	2.16	Interleukine-Natural killer cell stimulatory factor 1	NF-κB & IL-6 Signaling
19	TNFAIP	25.39	26.59	5.04	Tumor necrosis factor, ?-induced protein 3	Anti-inflammation
20	NFKBI	25.15	24.08	1.94	Nuclear factor of kappa light polypeptide gene	Transcription regulator
21	ATP2A2	24.00	30.09	53.82	ATPase, Ca++ transporting, cardiac muscle	Enzyme
22	9230106B05	13.48	27.43	119.10	RIKEN cDNA 9230106Bo5 gene	Enzyme
23	UBE2	10.41	4.56	32.53	Ubiquitin conjugating enzyme E 2A	Enzyme
24	ABCC	10.11	3.67	27.22	ATP-binding cassette, subfamily C	Protein
25	PKI	3.87	5.93	29.40	Protein Kinase (cAMP-dependent catalytic inhibi)	Enzyme
						

	**Ageing**	**Genes Down-regulated by LPS**				

**#**	**Genes**	**LPS**	**LPS + Dexa**	**Dexamethasone**	**Description**	**function**

1	ABCD2	-43.36	-2.06	4.69	ATP-binding cassette, subfamily D member 2	Protein
2	RASGRP3	-36.19	-46.69	-1.39	RAS guanyl releasing protein 3 (GRP3)	Protein
3	PTBP2	-32.63	-11.47	-6.69	Polypyrimidine tract binding protein 2	Protein
4	IFNGR1	-28.05	-16.15	-2.4	Interferon gamma receptor 1	Signaling
5	TNP1	-24.67	-13.06	-2.92	Transition protein 1	Protein
6	4930503B16	-22.71	-6.49	-2.08	RIKEN cDNA gene	Enzyme
7	PHKA2	-22.65	-7.54	3.32	Phosphorylase kinase, alpha 2 (liver)	Enzyme
8	UBE2D2	-22.6	-10.76	-18.83	Ubiquitin conjugating enzyme E2D2	Enzyme
9	HCAP-G	-22.08	-3.55	-4.89	Chromosome condensation protein G	Protein
10	ATP6V1H	-1.52	-1.36	-18.75	ATPase, H+ trrransporting lysosomal V1	Transport
11	LRRN1	-1.51	-500	-12.78	Leucine rich repeat neuronal 1	Enzyme
12	PRKGII	-1.39	-5.34	-17.23	Protein kinase, cGMP-dependent type II	Kinase

Out of several genes up-regulated or down-regulated with quercetin, 22 were associated with inflammation, and ageing process, which include gene expression of TRAF1, CCL8, VCAM1, RASGRP1, TNF-α, IL-1α, ABCC, and LRRN6A etc, which function in signaling, transcription, NF-κB, chemokine, protein synthesis, and only 10 genes down-regulated were associated with ageing process, such as ABCT, RASGRP3, PHKA, UBE2D and RIKEN cDNA were modulated by quercetin significantly (Table 8). The complete description and function of above mentioned genes is reported in Table 8.

Similarly, there were only 21 similar genes as reported for quercetin that were up-regulated and 10 genes down-regulated associated directly or indirectly with inflammation, and ageing process by δ-tocotrienol (Table 9). The genes whose expression was up-regulated by quercetin or δ-tocotrienol included Tgtp (T cell specific GTPase), TRAF1 (TNF receptor associated factor 1), VCAM1 (vascular cell adhesion molecule 1), ABCC (ATP-binding cassette, subfamily C), IL-1α (interleukin-1α), and gene expression down-regulated by quercetin and δ-tocotrienol included RASGRP3 (RAS guanyl releasing protein 3), IFBGR1 (interferon gamma receptor 1), PHKA2 (phosphorylase kinase-α2), UBE2D (ubiquitin conjugating enzyme E2D2) and RIKEN cDNA (Table 9).

The expression of 25 genes were up-regulated and 12 genes down-regulated responsible for the modulation of inflammation, and ageing process, which were impacted by dexamethasone treatment (Table 10). The important genes up-regulated with dexamethasone were CD40 (CD40 antigen belongs to TNF receptor superfamily), USP9 (ubiquitin specific protease 9), IL-1RA (interleukin 1 receptor antagonist), VCAM1 (vascular cell adhesion molecule), CCL8 (chemokine [C-C motif] ligand 8), NF-κB1 (nuclear factor of kappa B light polypeptide gene), PKI (protein kinase inhibitor), and RIKEN cDNA (9230106B05 gene) as shown in Table 10. The most important genes that were down-regulated due to dexamethasone treatment were ABCD (ATP-binding protein), RASGRP3 (ras guanyl releasing protein 3), PHKA2 (phosphorylase kinase, alpha2) and LRRN1 (leucine rich repeat neuronal 1), as shown in Table 10.

All the genes that were either up-regulated or down-regulated by quercetin, δ-tocotrienol, or dexamethasone described above were associated with transcription regulators, transcription receptors, cytokines, and kinases (Tables 8, 9, and 10). The maximum number of gene expression modulated by above mentioned inhibitors also influenced lipid metabolism, immune responses, sterol and bile acid biosynthesis, catalytic activity, blood coagulation, oxidoreductase activities, phosphotransferase activities, antigen processing, metal ion binding, RNA binding, and nucleic acid binding proteins (Tables 8, 9, and 10). In summary, the present preliminary results from the DNA-array followed by pathway analyses have shown that quercetin, δ-tocotrienol or dexamethasone inhibit LPS-stimulated gene expression at numerous points of the signaling pathways. The up-regulation and down-regulation of different genes in the pathway with each treatment is presented in Tables 8, 9, and 10. These results show that LPS up-regulates expression of several important genes associated with ageing and pro-inflammation (CCL8, NF-κB1, VCAM1, PK1, RIKEN cDNA, PHKA, IFBGR, LRRN, IL-1β, IL-1α, IL-6, TNF-α, IL-12, iNOS, VCAM1, ICAM1, COX2, IL1RA, TRAF1, CD40) and whose expression was inhibited by quercetin and δ-tocotrienol. The present results also suggest that approximately 90% of LPS-inducible macrophage genes are inhibited by quercetin, δ-tocotrienol and dexamethasone.

## Discussion

The key finding of the present study was that diet supplementation with quercetin or δ-tocotrienol for 4 weeks resulted in decreased expression levels of mRNA for TNF-α and iNOS genes and decreased secretion of TNF-α protein and production of NO by LPS-stimulated macrophages from both senescent and young mice. Stimulation of macrophages with either IFN-β or IFN-γ in addition to LPS significantly and markedly enhanced NO production compared to stimulation with LPS alone. Again, however, diet supplementation with quercetin or δ-tocotrienol for 4 weeks suppressed NO production by macrophages from both senescent and young mice when stimulated with LPS and either IFN-β or IFN-γ. We also demonstrated that NO levels and iNOS mRNA expression levels were significantly higher in LPS-stimulated macrophages from senescent, compared to young mice. In contrast, age did not appear to impact levels of TNF-α protein or mRNA expression levels in LPS-stimulated macrophages (Table 3).

Results of the current study of diet supplementation with quercetin and δ-tocotrienol confirm and extend our earlier findings that these same compounds inhibited NO production and TNF-α secretion by LPS-stimulated murine macrophages *in vitro*, and that serum NO and TNF-α levels were reduced in chickens fed diets supplemented with quercetin or δ-tocotrienol [3,5]. In view of evidence linking dysregulated inflammatory responses to a variety of age-associated diseases (e.g. cancer, cardiovascular disease, dementia), our collective findings that diet supplementation with two widely available, inexpensive, non-toxic proteasome inhibitors suppress inflammatory responses in mice and chickens, particularly in senescence, raises the prospect that diet supplementation with these agents could have beneficial health effects in ageing humans.

Quercetin and δ-tocotrienol are common, naturally occurring compounds as reported in detail in our recent publication [5], are commercially available as dietary supplements, and possess antioxidant, antithrombotic, anticarcinogenic and anti-inflammatory properties [8,16,17]. Quercetin and δ-tocotrienol have been shown to inhibit macrophage proliferation and activation *in vitro *by blocking the activation of LPS-stimulated NF-κB signaling [18-22]. Their inhibitory effect on the production of nitric oxide (NO) has been attributed to their antioxidant and free-radical scavenging properties [23,24]. Recently, we have described the detailed mechanism by which these compounds regulate the production of NO and pro-inflammatory cytokines [3]. We reported that proteasome inhibition by quercetin and δ-tocotrienol results in decreased proteolytic degradation of P-IκB which, in turn, decreases translocation of activated NFκB to the nucleus, and depresses transcription of TNF-α and iNOS genes [3]. These compounds appear to be relatively potent inhibitors of multiple proteasome subunits that are critically engaged in regulating inflammatory mediator/s in ageing processes [3].

The microarray data of quercetin and δ-tocotrienol support the involvement NF-κB and TLRs pathways for the regulation of production of nitric oxide as reported in Tables 4 - 10. The results from the DNA-array followed by pathway analyses have shown that quercetin, δ-tocotrienol, inhibits LPS-stimulated gene expression at numerous points. The up-regulation and down-regulation of different genes in the pathway from each treatment are presented in Tables 8 - 10. These results showed LPS-induced up-regulation or down-regulation of expression of several important genes associated with ageing and pro-inflammatory (NF-κB1, VCAM1, PKI, RIKEN cDNA, PHKA, LRRN, IL-1β, IL-1α, IL-6, TNF-α, IL-12, iNOS, ICAM1, IL1RA, TRAF1 and CD40) were inhibited by quercetin, δ-tocotrienol and dexamethasone. The present results also suggest that approximately 70% - 90% of LPS-stimulated macrophage genes were inhibited by quercetin and δ-tocotrienol. Therefore, effect of these compounds on many of the pathways identified by such studies combined use of the gene-chip array, coupled with the Ingenuity Pathway Analysis may provide definite information for identifying additional novel signaling pathways that are affected positively or negatively by these compounds (Tables 4 - 10).

## Conclusions

Recently, a great number of plant-derived substances have been evaluated as possible inhibitors of the NF-κB pathway. NF-κB is activated by pro-inflammatory stimuli such as TNF-α, LPS, and controls the expression of genes encoding the pro-inflammatory cytokine (TNF-α), as well as production of NO and iNOS enzyme. The majority of these compounds are antioxidants and act by blocking the phosphorylated and ubiquitinated IκB degradation by the proteasome, thereby preventing NF-κB activation. The results from the DNA-array followed by pathway analyses have shown that quercetin and δ-tocotrienol inhibit LPS-stimulated gene expression at numerous points. These results showed LPS-induced up-regulation or down-regulation of expression of several important genes associated with ageing and pro-inflammatory (NF-κB1, VCAM1, PKI, RIKEN cDNA, PHKA, LRRN, IL-1α, IL-6, TNF-α, IL-12, iNOS, ICAM1, IL-1RA, and TRAF1) mediators were inhibited by quercetin and δ-tocotrienol. The present findings of inhibition of nitric oxide production by quercetin and δ-tocotrienol may be of clinical significance in host defense mechanisms against various infections, treating several inflammatory diseases, including ageing process. Therefore, the evaluation of the molecular mechanisms of natural products-induced anti-inflammatory effects may provide novel means to regulate cellular function and to control NF-κB-dependent gene expression for therapeutic purposes, and such a new approach towards the role of nitric oxide in cellular signaling may lead to improved treatments for major diseases, including cardiovascular disease, diabetes, cancer, ageing and neurodegenerative disorders [25].

## Abbreviations

LPS: lipopolysaccharide; TNF-α: tumor necrosis factor-α; IL-1β: interleukin-1β; IL-1α: interleukin-1α; IL-6: interleukin-6; NO: nitric oxide; iNOS: nitric oxide synthase; NF-κB: nuclear factor-kappaB; IκB: inhibitor kappaB; TLRs: toll-like receptors; 1. CD: control diet; 2. Quer: quercetin; 3. Trie: δ-tocotrienol; 4. Dexa: dexamethasone.

## Competing interests

The authors declare that they have no competing interests.

## Authors' contributions

All the authors were involved in the designing of the study. Dr. XT (Postdoctoral fellow M.D.) carried out TNF-α, NO, and gene expression assays for this study. Ms. JCR (Post graduate student) carried out LPS-stimulated plus IFN-β and IFN-γ assays under the supervision of Drs. MZB, and NQ. Dr. CJP edited the manuscript. All the authors have read and approved the final version of the manuscript.
